# A Case Series of Intermittent Theta Burst Stimulation Treatment for Depressive Symptoms in Individuals with Autistic Spectrum Disorder: Real World TMS Study in the Tokyo Metropolitan Area

**DOI:** 10.3390/jpm13010145

**Published:** 2023-01-11

**Authors:** Yoshihiro Noda, Kyoshiro Fujii, Yu Mimura, Keita Taniguchi, Shinichiro Nakajima, Ryosuke Kitahata

**Affiliations:** 1 Department of Neuropsychiatry, Keio University School of Medicine, Tokyo 160-8582, Japan; 2 Shinjuku-Yoyogi Mental Lab Clinic, Tokyo 151-0051, Japan

**Keywords:** autistic spectrum disorder, depression, transcranial magnetic stimulation, theta-burst stimulation

## Abstract

Autism spectrum disorder (ASD) is a neurodevelopmental disorder characterized by deficits in social communication and the presence of restricted interests and repetitive behaviors. While the symptoms of ASD are present from early childhood, there has been an increase in the number of adults with ASD in recent years who visit healthcare professionals to seek the treatment of depression due to maladjustment resulting from the core symptoms and are eventually diagnosed with ASD. Currently, no treatment is available for the core symptoms of ASD, and pharmacotherapy and psychotherapy are often provided mainly for secondary disorders such as depression and anxiety. However, the effectiveness of these therapies is often limited in individuals with ASD compared to those with major depression. In this context, neuromodulation therapies such as transcranial magnetic stimulation (TMS) have gained increasing attention as potential treatments. In this case series, we retrospectively analyzed 18 cases with ASD from the TMS registry data who had failed to improve depressive symptoms with pharmacotherapy and were treated with intermittent theta burst stimulation (iTBS) therapy to the left dorsolateral prefrontal cortex (DLPFC). We also explored the relationship between treatment efficacy and clinical epidemiological profile. Our results indicated that, despite the limitations of an open-label preliminary case series, TMS therapy in the form of iTBS may have some beneficial therapeutic effects on depressive symptoms in individuals with ASD. The present findings warrant further validation through randomized, sham-controlled trials with larger sample sizes.

## 1. Introduction

Autism spectrum disorder (ASD) is a neurodevelopmental disorder that has been increasing in prevalence worldwide, including in Japan, in recent years [1,2]. Currently, no effective treatments exist for the core symptoms of ASD, such as qualitative impairments in communication and repetitive modes of interest, and the treatment options for individuals with ASD are limited to pharmacotherapy and psychosocial approaches [3,4]. In this context, non-pharmacological and non-invasive therapeutic approaches such as neuromodulation, especially the clinical application of transcranial magnetic stimulation (TMS), have recently gained a great deal of attention in the field of psychiatry [5,6]. Given the limited treatment options for ASD, the development of novel therapeutic approaches is urgently needed.

Individuals with ASD often exhibit symptoms of depression and anxiety resulting from maladaptation caused by the core symptoms [7]. While pharmacotherapy is often used with the aim of alleviating these symptoms, it may not always be successful and can cause unwanted side effects that often reduce the tolerance to medications. In addition, interpersonal problems and environmental adjustment problems caused by the core symptoms are often addressed through disease education and psychosocial approaches, but these strategies take time and have limited effectiveness. Furthermore, given the nature of ASD characteristics, the core symptoms may not even be considered as a target for treatment.

In light of the limitations of current treatments for secondary disorders of ASD, neuromodulation therapy, as represented by TMS, has emerged as a promising therapeutic strategy that psychiatrists can use to address these symptoms in the short term. Most of the previous studies have largely focused on the effectiveness of conventional TMS therapy in modifying the core symptoms specific to ASD [8,9]. However, few studies have evaluated its potential to target secondary disorders, including depressive symptoms, as a symptom target that is more readily modifiable by TMS intervention.

The objective of this case series was to preliminarily examine the efficacy of intermittent theta-burst stimulation (iTBS) treatment for depressive symptoms as a secondary disorder in individuals with ASD. We retrospectively surveyed the TMS registry data in the real world setting and extracted data for 18 patients with ASD. Specifically, Siddiqi and colleagues have recently shown that depression symptom networks form the common substrate, regardless of the underlying pathologies or intervention methods [10]. Based on this finding, we hypothesized that secondary depressive symptoms in individuals with ASD would have a comparable symptom network and that TMS treatment administered to the left dorsolateral prefrontal cortex (DLPFC) in individuals with ASD would exert some antidepressant effect. Hence, we analyzed the relevant clinical data in the TMS registry to test the hypothesis.

## 2. Materials and Methods

### 2.1. Case Series Setting

This study was conducted as a case series between 28 January 2020 and 30 November 2022, as part of the real world TMS registry study (jRCT1050210059) [11] at the Shinjuku-Yoyogi Mental Lab Clinic in Tokyo. The study included 18 outpatients with ASD who presented with depressive symptoms and met the following eligibility criteria. In principle, no changes in the pharmacotherapy regimen were made during the period of TMS therapy.

### 2.2. Eligibility Criteria

The eligibility criteria for this case series are as follows:

(1) Eighteen years of age or older;
(2) Patients who met the Diagnostic and Statistical Manual of Mental Disorders, 5th edition (DSM-5) definition of the diagnosis of ASD by consultation with a board-certified psychiatrist;
(3) Patients who had obvious depressive symptoms on psychiatric examination;
(4) Patients who had not achieved satisfactory improvement with pharmacotherapy, including antidepressants;
(5) Patients with the MADRS score of 15 or higher and moderate or higher depressive symptoms;
(6) Patients with no previous history of convulsive seizures;
(7) Patients with no other apparent contraindications to TMS therapy;
(8) Patients who had received a minimum of 15 sessions of TMS treatment and clinical assessment up to at least the interim time point.

### 2.3. Clinical Measures

Since this case series focused on the treatment of depressive symptoms, the severity of depressive symptoms was assessed using the Hamilton Depression Rating Scale—21 items (HAM-D_21_) and the Montgomery–Åsberg Depression Rating Scale (MADRS) as primary and secondary outcome measures, respectively. Clinical assessments were conducted at three time points: at baseline, prior to the start of TMS treatment; at an interim time point after a total of 15 sessions; and at a final time point after a total of 30 sessions. If the depressive symptoms reached remission or if there was no noticeable change in symptoms at an interim time point of the assessment, TMS treatment was terminated after a total of 15 sessions of TMS treatment after consultation with the patient.

### 2.4. TMS Treatment Protocol for the Depressive Symptoms in Individuals with ASD

The iTBS protocol, which has proven its usefulness in the THREE-D study [12], was used. Specifically, the iTBS protocol (a total of 20 trains of 50 Hz triplet bursts stimulated at a 5 Hz rhythm for 2 s and interrupted for 8 s, for a total of 600 pulses) was applied to the left DLPFC for approximately 3 min. The Beam_F3 method [13] was used to identify the stimulation site in each patient individually. The treatment course was based on a total of 30 sessions, with an interim evaluation at the end of 15 sessions. If remission was achieved at the interim evaluation or if the treatment was virtually ineffective, the treatment was terminated at that point.

### 2.5. Statistical Analysis

Since this case series was a preliminary open-label trial, paired *t*-tests were used to examine the changes in pre- and post-treatment scores on each measure under the assumption that there would be a therapeutic effect on depressive symptoms in individuals with ASD. Furthermore, we explored the correlations between clinical epidemiological data and the percent changes in each test score using Pearson’s correlation coefficients. The significance level was set at 0.05 in this study.

## 3. Results

A total of 18 individuals with ASD were included in this case series. These included 13 males and 5 females with a mean age (±S.D.) of 41.8 (±11.9) years. Detailed clinical epidemiological data and medication information at the time of TMS therapy introduction are summarized in Table 1 and Table 2. The mean stimulus intensity of TMS was 56.4 (±6.2)%, and all cases were performed at 120% RMT stimulus intensity. Eleven of the 18 patients received up to a total of 30 sessions of TMS therapy, and the remaining 7 patients received up to a total of 15 sessions of TMS therapy. Of the 7 patients who completed 15 sessions of treatment, 4 had remission of depressive symptoms, and treatment was terminated at that point, while the remaining 3 had no substantial change in symptoms, and treatment was discontinued at the halfway point.

**Figure 1:**
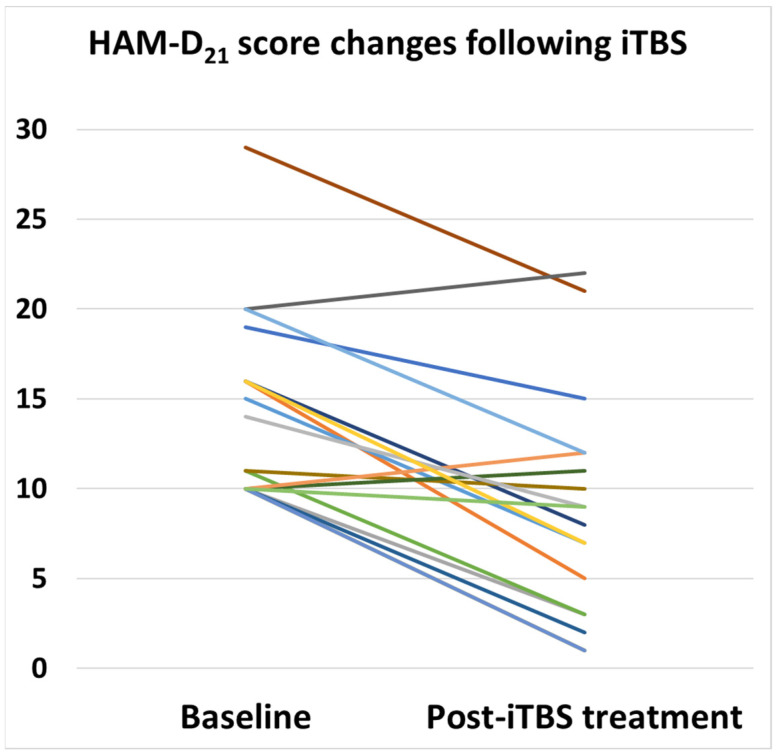
Longitudinal changes in HAM-D 21-item score following iTBS treatment for depressive symptoms in patients with ASD. Each color represents the score change for each patient.

The HAM-D score improved from 14.3 (±5.2) to 8.6 (±5.7) and the MADRS score improved from 23.7 (±6.3) to 14.2 (±10.2) following iTBS treatment (see Table 3). In terms of the primary outcome, the HAM-D score, 15 of the 18 patients showed an improvement in their scores, while 3 patients showed a mild worsening of their scores (Figure 1). With respect to the primary outcome, the response rate was 50% and the remission rate was 67%. On the other hand, when evaluated by the secondary outcome of the MADRS score, the response and remission rates were 44% and 33%, respectively, which were relatively low compared to the treatment outcome evaluated by the HAM-D score. AS for side effects, one patient (5.6%) complained of stimulation site pain, and no other obvious side effects or adverse events were observed in this case series.

In addition, there were no significant sex differences (HAM-D_21_ score changes: t_16_ = 1.253, *p* = 0.228; MADRS score changes: t_16_ = 0.995, *p* = 0.335) on the antidepressant effect in TMS treatment. Also, there was no significant relationship between the antidepressant effect of TMS treatment and age (HAM-D_21_ score changes: r = −0.064, *p* = 0.800, n = 18; MADRS score changes: r = 0.033, *p* = 0.895, n = 18). Furthermore, no significant correlation was found between treatment effect and stimulus intensity (HAM-D_21_ score changes: r = −0.370, *p* = 0.130, n = 18; MADRS score changes: r = 0.446, *p* = 0.063, n = 18).

## 4. Discussion

In this case series, iTBS treatment targeting the left DLPFC, which is an established treatment for depression, was administered to individuals with ASD who presented depressive symptoms. This treatment resulted in a response rate of 50% and a remission rate of 67% on the HAM-D score, and a response rate of 44% and a remission rate of 33% on the MADRS score, indicating a certain level of efficacy. These treatment outcomes were comparable to those achieved with conventional rTMS and iTBS therapy for treatment-resistant depression [14,15]. These findings suggest that neuromodulatory intervention with TMS for depressive networks may have antidepressant effects that are independent of the underlying psychiatric disorders and/or pathological conditions [10].

Furthermore, although the sample size in this case series is too small, the exploratory analyses did not find any significant effects of age or sex on the antidepressant effect of iTBS in individuals with ASD. Further research with a larger sample size is needed to examine the potential impact of benzodiazepine use on the antidepressant effect of TMS therapy, including iTBS for individuals with ASD, as benzodiazepines may reduce the therapeutic effect of TMS in the treatment of depression [16,17,18,19]. Additionally, as for sex differences, while males are overrepresented in the epidemiology of ASD, the proportion of male cases was more than double that of females in this case series. More research is needed to evaluate the potential impact of sex differences on the efficacy of TMS therapy in individuals with ASD in the future.

On the other hand, in terms of tolerability and adverse events, only one of the 18 individuals in this case series complained of stimulation site pain associated with iTBS treatment. This suggests that the increased sensitivity due to hypersensitivity often seen in individuals with ASD may not have a significant impact on tolerability or the occurrence of adverse events during iTBS treatment.

In addition, three patients showed a mild worsening of depressive symptoms following iTBS treatment. However, these three cases shared common psychosocial influences, such as maladjustment to the work environment and family conflict, during the treatment period. Thus, as with considerations during the introduction and administration of TMS therapy for major depressive disorder, the presence of such negative psychosocial modifiers should be carefully assessed prior to introducing TMS treatment in individuals with ASD. If such negative modifiers for depression are prominent, it may be advisable to withhold the introduction of TMS treatment.

Limitations of this case series include the following: First, since this case series was an analysis of the TMS registry data in the real world setting, it is limited in that it relies on the clinical diagnosis of ASD by psychiatrists based on the DSM-5 criteria. It may be beneficial to incorporate more precise assessment tools, such as the Autism Diagnostic Observation Schedule Second Edition, to ensure more accurate diagnoses. Furthermore, in Japan, TMS therapy for individuals with ASD is currently not officially provided, with limited practice using the iTBS protocol due to logistics at a few private TMS clinics in the Tokyo Metropolitan area. Thus, as more appropriate indications for TMS treatment for individuals with ASD are approved in the future, it will be possible to further increase the sample size and compare the usefulness of iTBS and high-frequency rTMS protocols. Moreover, in Japan, TMS therapy for treatment-resistant major depressive disorder is covered by public medical insurance, but in other cases, such as “adjustment disorder with depressed mood” based on ASD, most of those patients have depressive symptoms but only at a mild to moderate level. Therefore, the results of this case series of individuals with ASD based on TMS registry data reflecting the reality of TMS treatment in Japan may naturally differ from those of TMS therapy for patients with treatment-resistant major depressive disorder with moderate to severe depression because of the different backgrounds and settings. However, since the main objective of this case series was not to show the results of clinical research in which each condition was controlled as in a rigorous RCT study but to show the reality of TMS treatment in Japan as a real world study, this would be a meaningful report from a clinico-epidemiological perspective. Second, this case series has the limitation of a small sample size as it was a preliminary study using the recently launched TMS registry data. Hence, it will be important to replicate these findings in future studies with larger sample sizes to better establish the generalizability of these results. Specifically, a virtual comparison of the ASD and non-ASD groups using propensity score matching may allow us to examine the differential effects of TMS therapy in the two groups. Third, this case series only assessed the therapeutic effects of iTBS on depressive symptoms measured with the HAM-D_21_ and MADRS in individuals with ASD. However, it is important to note that the TMS treatment intervention in this study focused on secondary depressive symptoms rather than the core symptoms of ASD or social adjustment difficulties. Therefore, future TMS intervention studies should consider including assessments of the core symptoms and social adjustment levels due to ASD characteristics as primary outcomes. This will provide a more comprehensive understanding of the potential benefits of TMS in individuals with ASD. Fourth, due to the preliminary nature of this study and the lack of a control condition utilizing sham stimulation, the findings of this study should be validated through the use of randomized controlled trials in future research employing sham stimulation as a control. Finally, in this case series, the Beam_F3 method was used to identify the stimulation site for iTBS treatment. However, in the future, it may be better to apply more precise neuronavigation techniques based on individual MRI data.

## 5. Conclusions

The results of the current real world study using the TMS therapy-related registry data in Japan indicated that the FDA-approved iTBS protocol may have a favorable effect on reducing depressive symptoms in individuals with ASD. Further research with a larger sample size is needed to fully evaluate the efficacy, tolerability, and safety of TMS treatment, including iTBS, for the treatment of depressive symptoms in individuals with ASD.

## Figures and Tables

**Table 1:** Clinico-demographic information.

Characteristics	
Number of individuals; age: years (mean ± S.D.)	n = 18; 41.8 (±11.9)
Males	n = 13; 41.5 (±11.6)
Females	n = 5; 42.6 (±13.9)
Resting motor threshold (RMT), %	47.1 (±5.2)
Stimulus intensity for the left DLPFC (%MSO)	56.4 (±6.2) (120% RMT)

S.D.—standard deviation; DLPFC—dorsolateral prefrontal cortex; RMT—resting motor threshold; and MSO—maximum stimulator output.

**Table 2:** Medication information.

Case	Medication
1	Duloxetine 60 mg, Aripiprazole 15 mg, Flunitrazepam 2 mg, Eszopiclone 2 mg
2	Trazodone 150 mg, Fluvoxamine 150 mg, Olanzapine 10 mg, Zopiclone 10 mg
3	Sertraline 25 mg
4	-
5	Mirtazapine 45 mg, Lamotrigine 350 mg, Zopiclone 10 mg, Suvorexant 20 mg
6	Sertraline 100 mg
7	Duloxetine 30 mg, Zolpidem 10 mg
8	-
9	Duloxetine 60 mg, Aripiprazole 3 mg, Eszopiclone 3 mg
10	Duloxetine 40 mg
11	Duloxetine 60 mg, Escitalopram 10 mg, Aripiprazole 3 mg, Clonazepam 0.5 mg, Brotizolam 0.25 mg, Risperidone 0.5mg
12	Duloxetine 60 mg, Venlafaxine 75 mg, Aripiprazole 3 mg
13	Amitriptyline 50 mg, Paroxetine 37.5 mg
14	Fluvoxamine 150 mg, Aripiprazole 12 mg
15	-
16	Escitalopram 20 mg, Lemborexant 5 mg
17	Duloxetine 30 mg, Sertraline 25 mg, Flunitrazepam 2 mg, Suvorexant 20 mg
18	Etizolam 1.5 mg, Clotiazepam 15 mg

**Table 3:** Clinical outcome measures following TMS therapy.

Clinical Outcomes		Scores	Statistics
HAM-D 21-item score	pre-TMS (baseline) post-TMS	14.3 (±5.2) 8.6 (±5.7)	t_17_ = 5.514 *p* < 0.0001
MADRS score	pre-TMS (baseline) post-TMS	23.7 (±6.3) 14.2 (±10.2)	t_17_ = 4.153 *p* < 0.001

HAM-D—Hamilton Depression Rating Scale; MADRS—Montgomery–Åsberg Depression Rating Scale.

## Data Availability

All clinical data are available upon reasonable request to the corresponding author.
